# 3-D imaging reveals four extraordinary cases of convergent evolution of acoustic communication in crickets and allies (Insecta)

**DOI:** 10.1038/s41598-017-06840-6

**Published:** 2017-08-02

**Authors:** Laure Desutter-Grandcolas, Lauriane Jacquelin, Sylvain Hugel, Renaud Boistel, Romain Garrouste, Michel Henrotay, Ben H. Warren, Ioana C. Chintauan-Marquier, Patricia Nel, Philippe Grandcolas, André Nel

**Affiliations:** 1 Institut de Systématique, Évolution, Biodiversité, ISYEB - UMR 7205 – CNRS, MNHN, UPMC, EPHE, Muséum national d’Histoire naturelle, Sorbonne Universités, 57 rue Cuvier, CP 50, Entomologie, F-75005 Paris, France; 2 INCI, UPR 3212 CNRS, Université de Strasbourg, 5 rue Blaise Pascal, 67084 Strasbourg, France; 3 Université de Poitiers - UFR SFA, iPHEP UMR CNRS 7262, Bât B35 - TSA 51106, 6 rue Michel Brunet, F-86073 Poitiers Cedex 9, France; 4 Institute of Systematic Botany, University of Zurich, Zollikerstrasse 107, 8008 Zurich, Switzerland

## Abstract

When the same complex trait is exhibited by closely related species, a single evolutionary origin is frequently invoked. The complex stridulatory apparatus present in the forewings of extant crickets, mole crickets, katydids, and prophalangopsids, is currently interpreted as sharing a single common origin due to their similarity and unique function. An alternative hypothesis of convergent evolution in these ensiferan groups has challenged this common view, but remained controversial because of competing interpretations of wing venation. Here we propose another hypothesis for the widely and long debated homology of ensiferan stridulatory apparatus, performing the first 3D reconstruction of hidden structures at the wing bases. This approach allowed defining the homology of each vein from its very origin rather than after its more distal characteristics, which may be subjected to environmental pressure of selection. The stridulatory apparatus involves different veins in these four singing clades. In light of the most recent phylogenetic evidence, this apparatus developed four times in Ensifera, illustrating extraordinary convergent evolutions between closely related clades, by far exceeding the number of evolutionary steps ever proposed for calling ability in this group.

## Introduction

The Orthoptera Ensifera are well known for their capacity to emit and perceive sounds, having developed a high diversity of morphological structures and behaviors not commensurate with other animal groups^1^. Their sounds are produced by the friction between two sclerotized parts of the body (=stridulation), in two main modalities: a) wing stridulation, i.e., the friction between the two forewings (or tegmina), in crickets, prophalangopsids, katydids, and mole crickets; b) femoro-abdominal stridulation, i.e., the friction of a hind femora against the adjacent abdominal tergites^2, 3^. Vibratory or acoustic communication can be found in representatives of all the currently recognized ensiferan families. Yet efficient sound production by wing stridulation is amazingly complex needing several crucial biophysical and neurological couplings, this fact being counter-intuitive for convergence.

Ensiferan wing stridulation is based on intricate anatomical structures located on the veins of the basal part of the tegmina (venation terminology in Supporting Information). When the tegmina come to overlap by a lateral movement of opening - closing, the ventral face of one tegmen, bearing a row of teeth called the file, rubs the thickened rim (scraper) of the other tegmen^3^. The generated vibrations are amplified by a resonator located very close to the file and the scraper: a circular area (mirror) in katydids (Tettigonioidea), a triangular harp sustained by parallel and generally oblique veins in mole crickets (Gryllotalpoidea, Gryllotalpidae) and crickets (Grylloidea), or a variation of large wing cells in prophalangopsids (Hagloidea, Prophalangopsidae). The resonator multiplies the frequency and amplifies the intensity of the signal, and the vibrations propagated along the main veins^4^. In crickets, the resonator properties fit the closing speed of the wings, and both fit the dominant frequency of the emitted song, resulting in musical signals. *Cyphoderris* emits a highly-tuned signal, which contrasts with the signal most often broad-banded with a large ultrasonic part emitted by most katydids^5, 6^. In mole crickets, the efficiency of sound production increases when the male sings from inside its burrow^7^. The main controversy regarding acoustic origins concerns the homology of acoustic apparatus, which can be approximate through the nature of the file, carried by veins whose homology is discussed^2, 5, 8–12^. These studies lead to two alternative hypotheses for file location, on posterior cubital or first anal veins. The last study^12^ concluded in favor of a single origin of acoustic communication on the basis of the hypothesis that the file is located on CuP and homologous in all singing Ensifera.

Most recent attempts of establishment of the homology of these structures were based on the relative positions and relative convexity vs. concavity of the concerned cubital and anal veins^5, 12^, without exams of the extreme bases of the veins and their departures from the basivenal sclerites. The identity of each vein can be more accurately determined by examining from where it begins than from its relative position and relative convexity in distal parts, which are more subject to environmental pressure of selection. Traditionally the long and thin organs that constitute insect wings are intuitively simplified as a 2D structure and examined using optical microscopy. This technique is unable to separate strongly approximate or touching veins at the very base of the wings, as are the CuP and A1. Here we examine the relative positions of the veins in 3D space using X-ray microtomography (XMT): 3D modeling of venation, from the extreme bases of the veins to the functional acoustic apparatus, helps following each vein individually from its emergence from a basivenal sclerite, but also potential vein anastomoses, fusions and separations. We explored the diversity of wing venations in singing Ensifera (see Suppl. Table 1), to establish the homology of the stridulatory structures in crickets, mole crickets, prophalangopsids, and katydids.

For a better comparison with the last studies on the topic^5, 12^, we use the same nomenclature of wing venation elaborated by Béthoux & Nel^13^, adapted with slight modifications by Béthoux in subsequent papers^5, 12^. Corresponding abbreviations are: CuPa = anterior branch of CuP; CuPaα = anterior branch of CuPa; CuPaβ = posterior branch of CuPa; CuPb = posterior branch of CuP; first anal vein = A1. Abbreviation for the other veins and color codes are listed in the Supporting Information.

**Figure 1:**
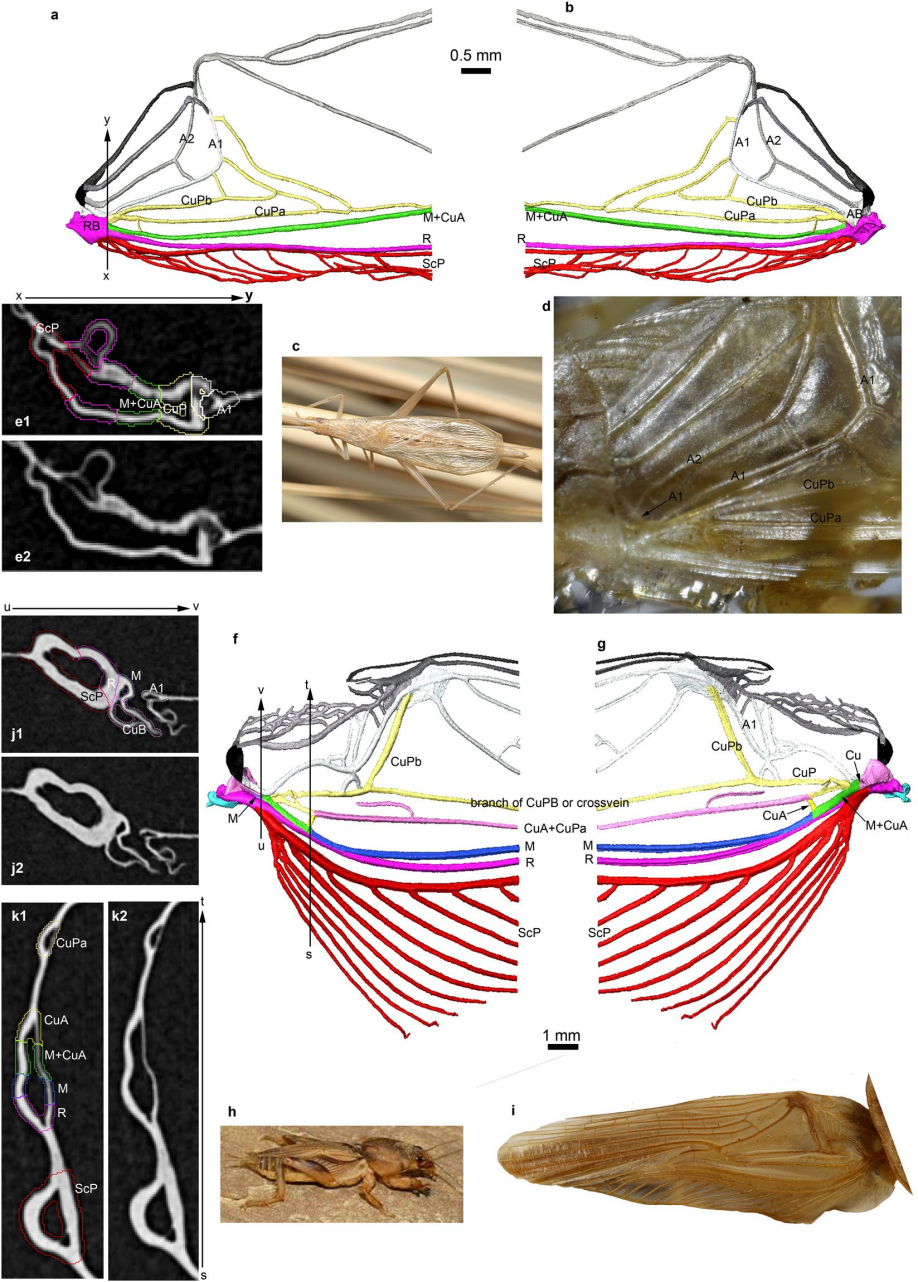
Wing venation interpretations. (**a**–**e**) Gryllidae (*Oecanthus* sp.). (**a**) 3D modeling of wing venation using XMT, view from above. (**b**) 3D modeling of wing venation using XMT, view from below. (**c**) General habitus (copyright S. Hugel). (**d**) Complete tegmen (copyright L. Desutter-Grandcolas). (**e**) Cut xy (1 colored; 2 without color; orientation of tomogram plane YZ, tomogram number 1035). (**f**–**k**) Gryllotalpidae (*Scapteriscus* sp.). (**f**) 3D modeling of wing venation using XMT, view from above. (**g**) 3D modeling of wing venation using XMT, view from below. (**h**) General habitus (copyright S. Hugel). (**i**) Complete tegmen (copyright L. Desutter-Grandcolas). (**j**) Cut uv (1 colored; 2 without color; orientation of tomogram plane XZ, tomogram number 1043). (**k**) Cut st (1 colored; 2 without color; orientation of tomogram plane XZ, tomogram number 876). (3D XMT L. Jacquelin).

XMT reconstructions show that the stridulatory file of the Grylloidea is on A1 (Fig. 1a–e, Supporting Information), while it is on CuPb in the Gryllotalpidae (Fig. 1f–k, Supporting Information). Consequently the triangular harp is an area between A1 and the most anterior branch CuPa of CuP in crickets, unlike the hypothesis of Béthoux^12^, while it is an area between the two branches CuPa and CuPb in mole crickets, in accordance with Béthoux^12^. Thus, the stridulatory files and harps of crickets and mole crickets are not homologous (Ref. 2, *contra* ^5, 8–12^), while they are currently considered as sister groups^14^. Béthoux^12^ proposed that in mole crickets CuA is separating from M very basally and fused with CuPa into a very long CuA+CuPa, unlike in Grylloidea. We recover a strong transverse vein between M+CuA and CuPa in the mole cricket that could correspond to the CuA.

**Figure 2:**
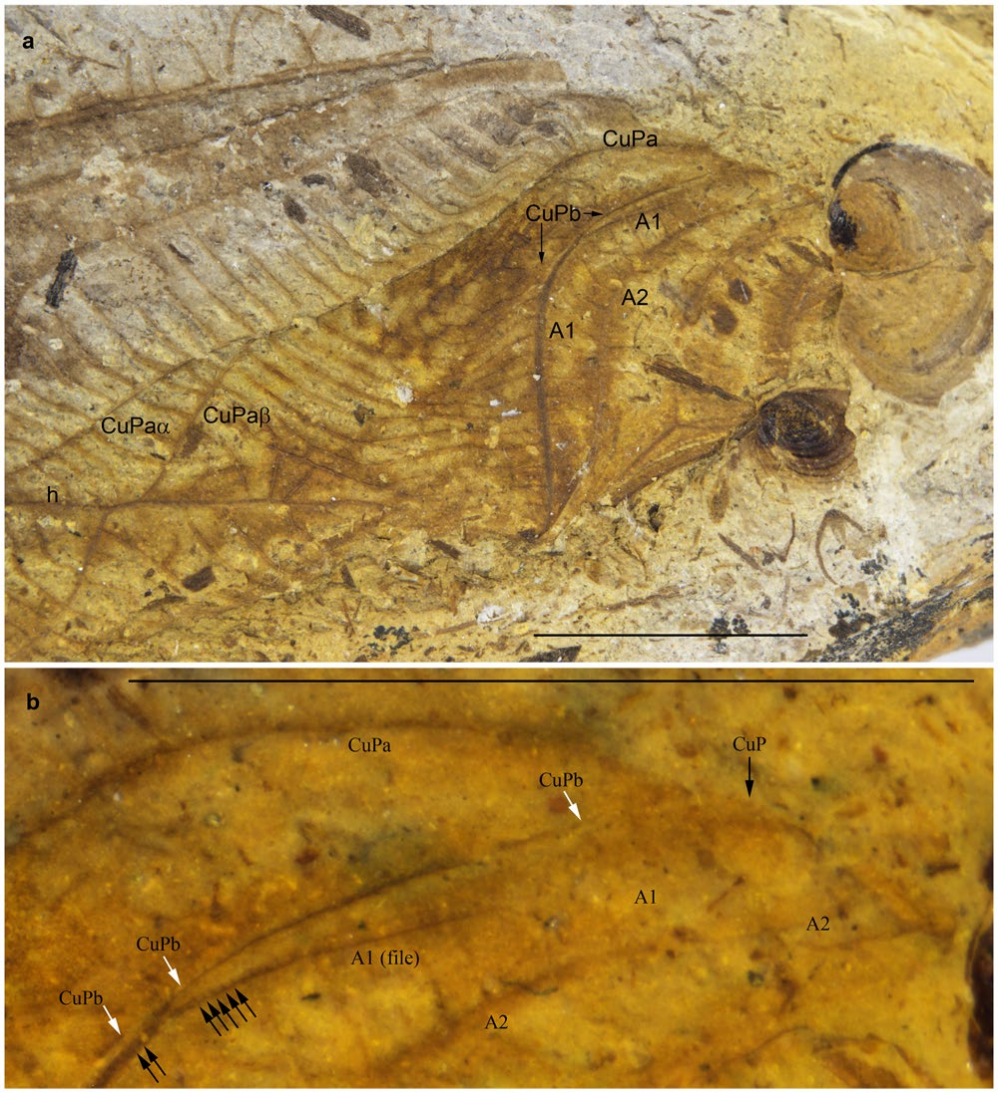
Wing venation interpretations. *Liassophyllum caii* Gu & Ren, 2012, holotype CNU-ORTNN2009008. (**a**) Wing base. (**b**) Magnification of bases of CuP and anal vein. Series of black arrows: teeth of the file; white arrows: course of CuPb meeting A1 (file) in one point. Scale bars = 5 mm (copyright Dong Ren).

The Grylloidea are characterized by a very weak (sometimes completely reduced, especially in Gryllinae. s. str.) CuPb vanishing in area between CuPa and A1. Béthoux^12^ considered this vein as ‘a sclerotization located between CuPa and CuPb’, but this vein is clearly emerging from the cubital basivenal sclerite in all the Grylloidea we examined (see for example *Oecanthus* sp., where it is especially strong: Fig. 1a,b,d, suppl. movies 1,2). This vein is not homologous to the secondary structure present in *Gryllotalpa* between CuA+CuPa and CuPb, which is not emerging from a basivenal sclerite (Fig. 1f–k, suppl. movies 3,4). In the same line of evidence, the type forewing of the Jurassic *Liassophyllum caii* Gu & Ren, 2012 (currently in Haglidae: Cyrtophyllitinae) shows a short but well-defined CuPb emerging from the same stem as CuPa and only touching (but not fused with) the vein that bears the file teeth (Fig. 2). The file itself is not basally fused with Cu but joins the anal veins: it corresponds to A1. CuPb is distally vanishing in the area between the file and CuPa. The venation pattern of *Liassophyllum caii* does not fit the situation found in other Prophalangopsidae and Haglidae (clearly illustrated by the fossil prophalangopsid *Sigmaboilus peregrinus* Gu *et al*., 2009) (see Supplementary Fig. 1), and calls for the reexamination of the monophyly of the group Hagloidea, or the actual taxonomic identities of the fossils classified in this group.

**Figure 3:**
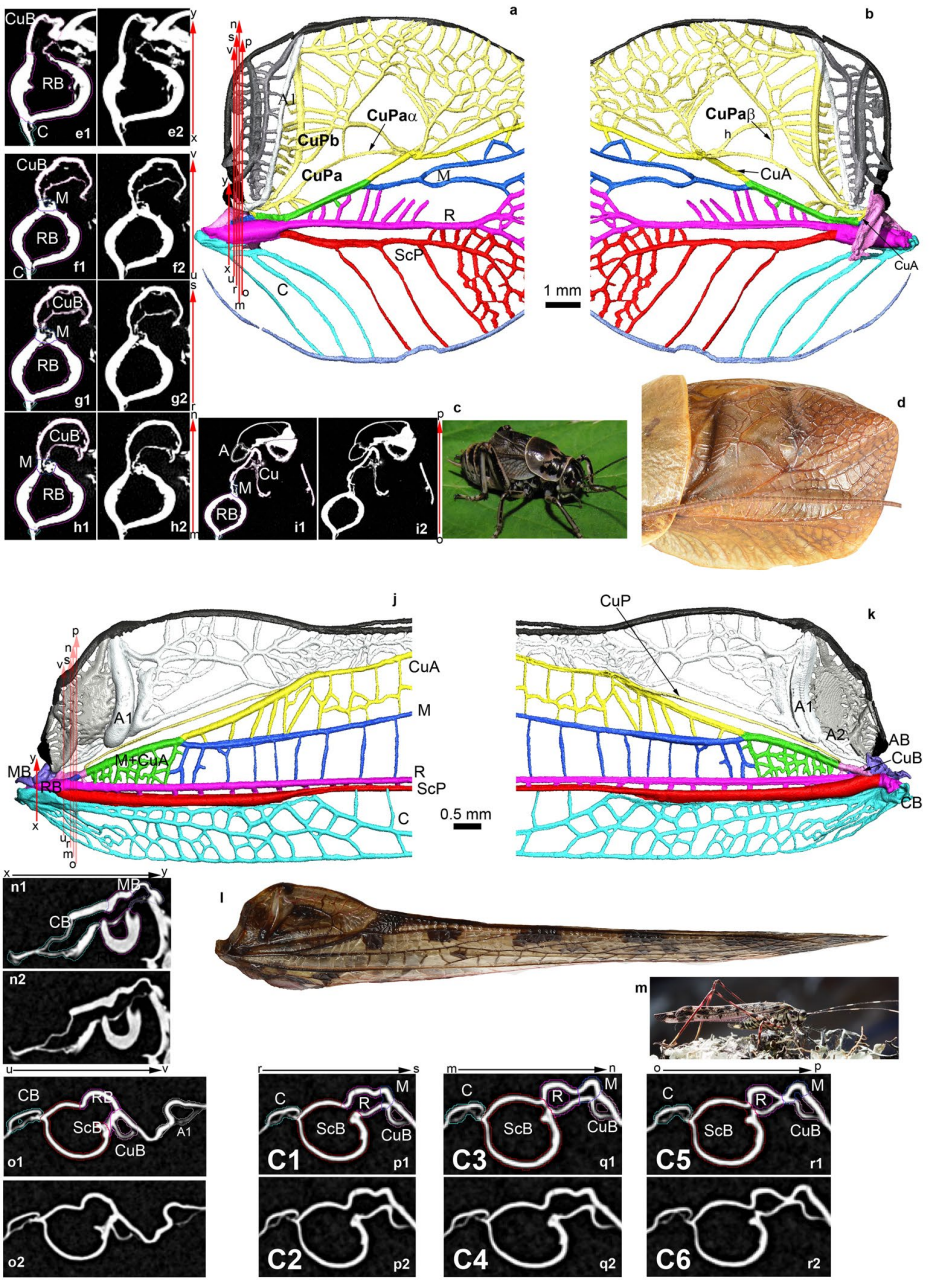
Wing venation interpretations. (**a**–**i**) Prophalangopsidae (*Cyphoderris monstrosa*). (**a**) 3D modeling of wing venation using XMT, view from above. (**b**) 3D modeling of wing venation using XMT, view from below. (**c**) General habitus (copyright and thanks to D. Gwynne for authorization to use). (**d**) Complete tegmen (copyright L. Desutter-Grandcolas). (**e**) Cut xy (1 colored; 2 without color; orientation of tomogram plane XY, tomogram number 1159). (**f**) Cut uv (1 colored; 2 without color; orientation of tomogram plane XY, tomogram number 1131). (**g**) Cut rs (1 colored; 2 without color; orientation of tomogram plane XY, tomogram number 1134). (**h**) Cut mn (1 colored; 2 without color; orientation of tomogram plane XY, tomogram number 1138). (**i**) Cut op (1 colored; 2 without color; orientation of tomogram plane XY, tomogram number 1112). (**j–o**) Tettigonioidea (*Quiva* sp.). (**j**) 3D modeling of wing venation using XMT, view from above. (**k**) 3D modeling of wing venation using XMT, view from below. (**l**) General habitus (copyright and thanks to P.S. Padron for authorization to use). (**m**) Complete tegmen (copyright L. Desutter-Grandcolas). (**n**) Cut xy (1 colored; 2 without color; orientation of tomogram plane XZ, tomogram number 130). (**o**) Cut uv (1 colored; 2 without color; orientation of tomogram plane XZ, tomogram number 207) (**p**) Cut rs (1 colored; 2 without color; orientation of tomogram plane XZ, tomogram number 223). (**q**) Cut mn (1 colored; 2 without color; orientation of tomogram plane XZ, tomogram number 232). (**r**) Cut op (1 colored; 2 without color; orientation of tomogram plane XZ, tomogram number 235). (3D XMT L. Jacquelin).

The stridulatory files of the modern prophalangopsids are on the concave CuPb as in the Gryllotalpidae (Fig. 3a–i, Supporting Information, suppl. movies 5,6). The main difference with the latter group is that, in the modern Prophalangopsidae, the branch CuPaβ of CuPa is separating from the anterior branch CuPaα basally far from the distal point of contact of CuP with CuA, and is ending in a strongly convex supplementary vein named handle (h)^12^, which seems to be proper to the Prophalangopsidae. In the Grylloidea, CuPaβ is separating from CuP at its point of separation with CuA (thus corresponding to a probable fusion of CuPaβ with h^12^; CuPaβ is ending in a node of veins situated at the posterior end of the file.

The stridulatory apparatus of the Tettigonioidea shows some similarity with that of the Grylloidea because the stridulatory file is clearly on A1 that emerges from the anal basivenal sclerite (Fig. 3j–r, suppl. movies 7,8). This interpretation is in accordance to that of Sharov, fig. 9E^15^, who considered a simple Cu vein (our CuP). This situation is completely different from those in Gryllotalpidae and modern Prophalangopsidae (Fig. 3a–i, Supporting Information). In addition, the stridulatory file and the A1 vein of the katydids show a unique configuration: A1 is convex but strongly flattened, and it is strongly connected to a more distal vein that emerges at the base of the stridulatory file, and is distally bifurcating. This vein looks like an anterior branch of A1, which is rather surprising because A1 is generally simple, although exceptions exist in the polyneopteran Phasmatodea (viz. *Achrioptera*, *Heteropteryx*) and in holometabolan Neuropterida^16^. This complex structure between the file and CuPa was interpreted as of composite origin (CuPaβ, columb, and handle) by Béthoux^12^ and Chivers *et al*.^5^. Furthermore in the katydids, CuPb is absent while the basal part of CuPa is weak and concave, and runs between the strongly convex A1 and the convex M+CuA. Therefore, even if the katydid and grylloid stridulatory files are on the vein A1, their whole stridulatory apparatus are not homologous.

From a functional point of view^3^, the file has to be situated on a strong relief of the ventral side of the tegmen to rub the other tegmen. Prophalangopsids and gryllotalpids have easily ‘solved’ the problem as the file is located on the ventral side of the concave vein CuPb (thus highly protruding on the ventral side of the wing and in a position to hit the scraper). In the grylloids, the file is on the distal part of vein A1, which is concave at the file level but convex basally (and thus changes its convexity, rendering its homology difficult to interpret on the sole criteria of the position and convexity of the vein). In the katydids, A1 is convex but strongly flattened so that its ventral part bearing the file is ventrally protruding.

**Figure 4:**
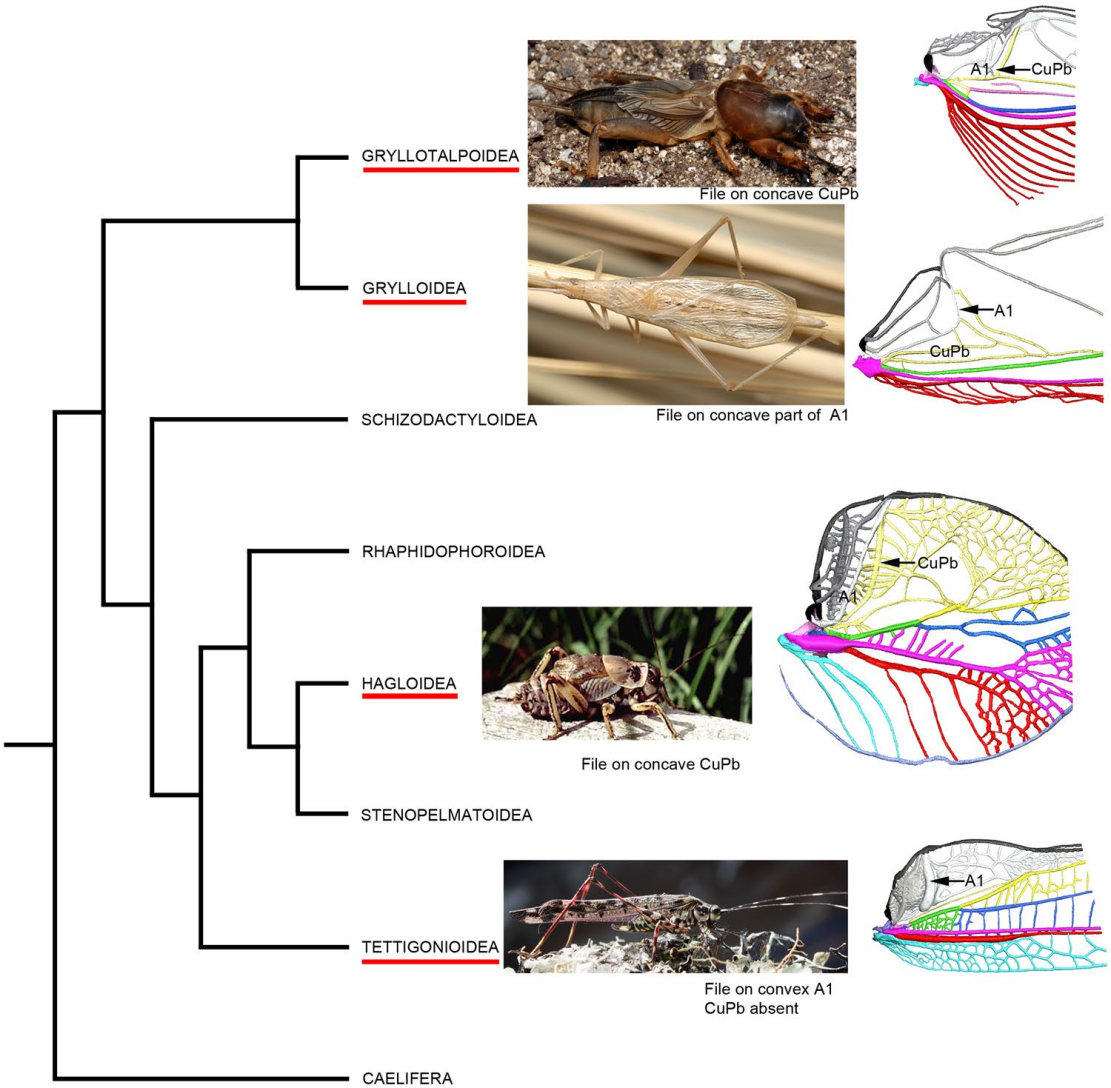
Molecular phylogeny of Ensifera (after Song *et al*.^12^). The four singing groups are indicated by photographs of representatives and the reconstructions of the cubito-anal fields (arrows indicate the files) (copyrights of photographs S. Hugel, D. Gwynne, P.S. Padron).

According to XMT results, a stridulatory file has evolved convergently in Ensifera, on the cubitus posterior vein of the mole crickets and prophalangopsids, and on the anal vein in crickets and katydids (Fig. 4), with different venation patterns each time. Following the phylogeny proposed by Song *et al*.^14^, with the crickets and mole crickets within Gryllidea, and the katydids and modern prophalangopsids within Tettigoniidea, the acoustic communication should have evolved independently at least four times in Ensifera. Our inference of convergence is strong enough compared to other homoplastic patterns with difficult probabilistic inference^17^: we demonstrate differences between wings that cannot be re-interpreted as a common ancestral pattern depending on their situation on a tree. Interestingly, the Permian Permostridulidae (Palaeozoic orthopteroid order Caloneurodea) have a stridulatory file on a supplementary longitudinal veinlet between CuPa and CuPb (Supplementary Fig. 2)^18, 19^. It represents a fifth type of acoustic apparatus developed on the forewings of an Archaeorthoptera. It is commonly accepted that acoustic communication is counter-selected by strong predation^20–22^. It can then be supposed that each emergence of acoustic communication could be related to a ‘low-predation window’, i.e. a period with less intense predation pressure.

From an evolutionary and functional point of view, because of the general pattern of convergences for stridulation put in evidence here in Ensifera, the functional parameters that govern the properties of emitted signals cannot be analyzed between ensiferan clades as deriving from supposedly ancestral groups to supposedly more apical ones, which would remind a gradist approach (*contra* ^5^): each clade evolved on its own under relatively similar functional constraints. This pattern invalidates the concept of ‘Grylloptera’ sensu^12^, a ‘formal taxon encompassing ensiferans possessing a ‘file’^5^ and a paraphyletic assemblage proposed without taking into account the actual diversity of the Ensifera. The only way to hypothesize a plesiomorphic condition for the whole Ensifera will be to perform a phylogenetic analysis including both extant and fossil species, both mute and acoustic, with well-attested homology hypotheses.

**Figure 5:**
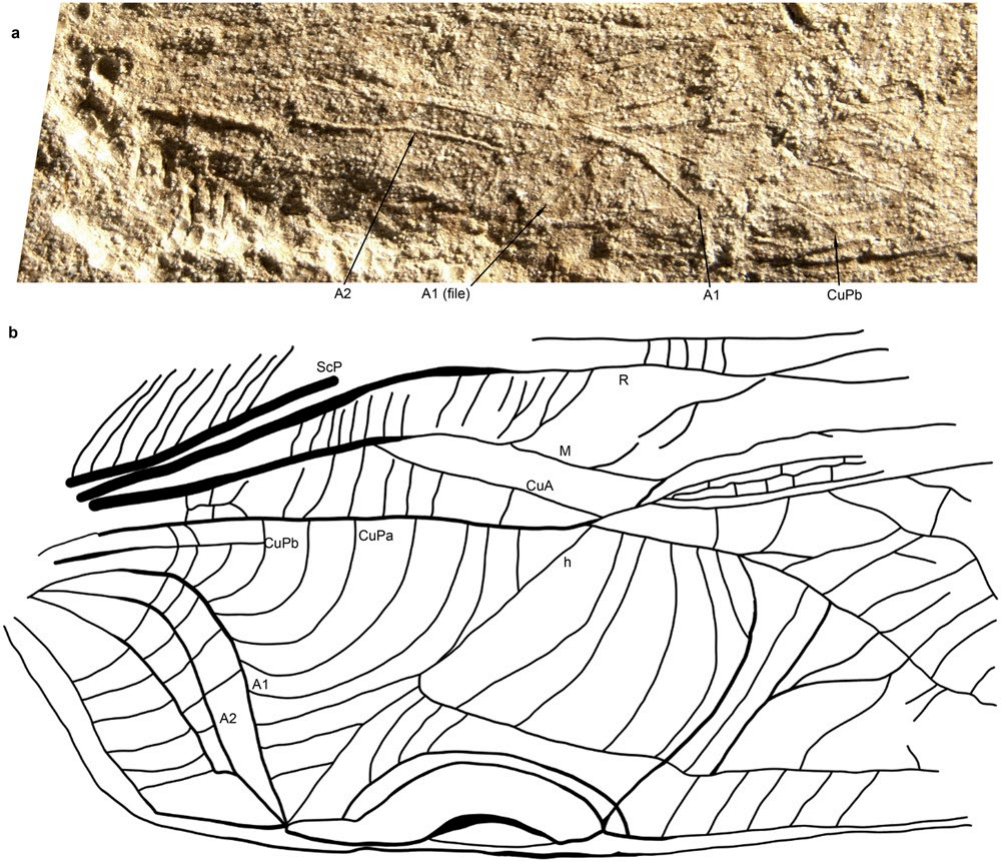
Wing venation interpretations. (**a**) *Mesoedischia obliqua* Gorochov, 1987, holotype PIN 2240/4074, Triassic, Kyrgyzstan, photograph (copyright A. Rasnitsyn); for the reconstruction of tegmen see ref. 28. (**b**) Reconstruction of tegment of a Grylloidea indet., MNHN.F.A57514, Torcian, Bascharage, Grand-Duché du Luxembourg (copyright A. Nel).

The majority of the Mesozoic singing Ensifera are currently attributed to fossil families (e.g., Mesoedischiidae Gorochov, 1987, Haglidae Handlirsch, 1906) or to the Prophalangopsidae, without strong supporting synapomorphies. Our new hypothesis for the homologies of the singing apparatus provides a new frame to reconsider their relationships. For instance, the Triassic *Mesoedischia obliqua* Gorochov, 1987 or *Termitidium ignotum* Westwood, 1854 could have a file patterns of tettigonioid type (see Fig. 5a). The recent description of a leaf-mimicking Tettigonioidea in the Middle Permian demonstrates the antiquity of this clade^14^ contra^23, 24^. The grylloid type is present in an undescribed Liassic fossil from Grand-Duché de Luxembourg (see Fig. 5b, Suppl. Fig. 3).

Beyond the necessary reanalysis of the venation of the other modern and fossil Ensifera in the light of the paradigm we validate here, several questions need to be addressed in the next future.

First, what are the molecular mechanisms underlying diversified wing venation in Ensifera? Second, are the same genomic regions responsible for more than one wing venation pattern? Third, are the same genes responsible for the same wing venation pattern for all the Ensifera? Turning on and off a key set of genes may have resulted in different wing patterns, by alteration of their expression regulatory pathway^25^, supporting the hypothesis that repeated reversals or convergences of characters are likely in the evolution of Ensifera. In this context, the exploration of genes and their expression by means of high throughput sequencing methods would enlighten us on the reversible and irreversible genetic changes driving morphogenesis in insects, over long periods of evolutionary time^26, 27^. In responding to questions such as these, the new framework we present here for understanding Ensiferan acoustics paves the way for a more detailed understanding of the processes giving rise to convergent evolutionary outcomes.

## Methods

### Materials

The studied specimens are housed at the Entomology Department of the Muséum national d’Histoire naturelle, Paris, France. The inventory numbers are detailed in Suppl. Table 2, see MNHN collection data base at https://science.mnhn.fr/institution/mnhn/collection/eo/).

### Imaging

The four extant specimens were imaged under X-ray, with phase contrast, at the microtomograph of the University of Poitiers.

## Electronic supplementary material

supplementary information

video 1

video 2

video 3

video 4

video 5

video 6

video 7

video 8
