# Supplementary material for: 3-D imaging reveals four extraordinary cases of convergent evolution of acoustic communication in crickets and allies (Insecta)
Source: Sci Rep. 2017 Aug 2;7:7099. doi: 10.1038/s41598-017-06840-6 (PMC5541040; doi:10.1038/s41598-017-06840-6)
Supplement: Supplementary file 1 — supplementary information [file 41598_2017_6840_MOESM1_ESM.pdf]

# 3-D imaging reveals four extraordinary cases of convergent evolution of acoustic communication in crickets and allies (Insecta)

Laure Desutter-Grandcolas[1], Lauriane Jacquelin[1], Sylvain Hugel[2], Renaud Boistel[3], Romain Garrouste[1], Michel Henrotay[1], Ben H. Warren[4], Ioana C. Chintauan-Marquier[1], Patricia Nel[1], Philippe Grandcolas[1], & André Nel[1]

SUPPLEMENTARY INFORMATION

## S.1. Supplementary methods

### 1.1. Studied material

Four male specimens of extant Ensifera were studied, one Grylloidea, (*Oecanthus* sp., Gryllidae: Oecanthinae); one Gryllotalpoidea (*Scapteriscus* sp., Gryllotalpidae); one Hagloidea (*Cyphoderris monstrosa* Uhler, 1864, Prophalangopsidae Cyphoderrinae); and one Tettigonioidea (*Quiva* sp., Tettigoniidae: Phaneropterinae). All specimens are in the entomology collection of the Muséum national d'Histoire naturelle (MNHN), all dried except for the Phaneropterinae, preserved in alcohol; inventory numbers are detailed in Suppl. Table 1, see MNHN collection data base at https://science.mnhn.fr/institution/mnhn/collection/eo/).

**1.2. Imaging**. The four specimens were imaged under X-ray, with phase contrast. XMT was performed according to the protocols reported in the literature[1-2-3]. We used a microtomograph RX solutions EasyTom XL Duo, using a Hamamatsu nanofocus160 kV 8W, source and a Varian-Paxscan 2520DX CsI detector, at the Centre for Microtomography of the University of

Poitiers (France). Scans were performed at 70 kV and 35-39 μA. The geometry was set to obtain a 5.44–8.83 μm voxel size in the reconstructed three-dimensional images. The reconstruction was performed using the FDK algorithms of Xact. 4.3. The dataset consists of 1440 projections taken over 360° for the wings of the specimen. For all the specimens, the left wing was separated from the body, cut largely above the wing sclerites in the pronotum. After it was placed between two blocks of emalen, except for the wing basis (with basivenal sclerites) that was placed out of emalen. To be scanned, each forewing was placed in a plastic tube, perpendicularly for *Cyphoderris* and in parallel for others at the beam of CT-scan. For each specimen, the image acquisition parameters are listed in Suppl. Table 1.

After the scan, it was necessary to increase contrast/brightness of tomograms in order to facilitate the distinction between the structures and to reduce the grey level values to 8-bits, i.e., to reduce the data size, and finally create a 'slice', an orderly stack of tomograms to an easy handling thereafter. For this scope, we used the ImageJ® (64-bit) software (Rasband, W.S., ImageJ, U.S. National Institutes of Health, Bethesda, Maryland, USA, http://imagej.nih.gov/ij/, 1997-2015).

3D volume rendering (Isosurface) was used to visualize the sub-set of selected voxels of the wings structure in AVIZO (FEI, Merignac, France, https://www.fei.com/software/amira-avizo/). This was performed using a manual segmentation tools (the paint brush) with a limitation of level of grey. A specific color was assigned for each homologous structure, viz., pale blue for CB and C, red for ScB and ScP, dark pink for RB and R, violet of MB, dark blue for M, green for M+CuA, pale pink for CuB and common stem of Cu, dark yellow for CuA, pale yellow for CuP, pale or dark grey for anal veins, black for AB(+JB). The segmentation process resulted for each dataset in a 'label' dataset with the same dimensions as the corresponding slice. The microtomography analyses show the wing membrane, the ribs and thoracic basal sclerites. For convenience, we have chosen to reproduce in 3D models the ribs,

basivenal sclerites, and most of the crossveins that were visible on tomograms although these were not essential for the study; therefore, the edges of wings generally do not appear, unless they are evidenced by a rib.

In order to complete the 3D modeling, each specimen was observed under a binocular microscope, model Olympus SZX9, with a target PLAPO 1X DF-2. The wing venations were photographed by a Nikon AF-S Micro camera 60 / 2.8G ED via the Camera Control Pro 2 software.

| **Taxon** | **Collection number** | **Accelerating voltage (KV)** | **Intensity (µA)** | **Numerisation angle** | **Number projections** | **Exposure time (s)** | **Voxel size (µm)** |
|---|---|---|---|---|---|---|---|
| Tettigoniidae (*Quiva* sp.) | MNHN-EO-ENSIF4012 | 70 | 39 | 360° | 1440 | 0.5 | 6.39 |
| Prophalangopsidae *Cyphoderris monstrosa* | MNHN-EO-ENSIF3908 | 70 | 35 | 360° | 1440 | 0.5 | 8.76 |
| Gryllidae *Oecanthus* sp. | MNHN-EO-ENSIF4013 | 70 | 39 | 360° | 1440 | 0.5 | 5.44 |
| Gryllotalpidae *Scapteriscus* sp. | MNHN-EO-ENSIF3068 | 70 | 39 | 360° | 1440 | 0.5 | 8.83 |

**Table 1. List of taxa and parameters for CT-scan segmentation**

### 1.3. Definitions of wing structures

Definitions are necessary for the understanding of the paper: veins are named and studied from the most anterior to the most posterior. Independently of the problems of homology, the names and abbreviations are as follows: C = costal vein; Sc = subcostal vein; R = radial vein; M = median vein; Cu = cubital vein; A = anal vein; J = jugal vein; XA = anterior branch of a vein X; XP posterior branch of a vein X.

**Basivenal sclerite**s: sclerotized blood cavities, at the wing base, by which the hemolymph arrives in the wing[4]. The basivenal sclerites are the most distal series of sclerites between the thorax and the wing[4-5]. They are named: CB costal basivenal; ScB subcostal basivenal; RB radial basivenal; MB median basivenal; CuB cubital basivenal; AB anal basivenal; JB jugal basivenal. The existence of this complete series has to be verified, as inconclusive results were obtained with the studied specimens (see below).

**Folds**: the wing membrane can be folded near or between the veins. Folds can be simple or branched, longitudinal or transverse. Some strong folds can be reinforced by thickenings of the membrane, called false veins (viz. pseudo-median vein of the Diptera Syrphidae)[3].

**Trachea**: tube of the respiratory system that can ramify into tracheoles, to bring the air to all the organs.

**Vein**: hollow tubular structure formed by the coupling of the upper and lower sheets of the wing membrane, supporting and consolidating it. The most important veins are longitudinal and can contain nerve, trachea, and hemolymph. The smaller, called crossveins, transverse, are stiffening structures. Veins can be thick or thin, broadened or narrowed, with an oval or subquadrangular section[6], corrugated or hollow[7].

**Vein concavity/convexity**: a concave vein (indicated by a ‘-’ in figures) is placed in low position on the ventral sheet of the wing membrane, while a convex vein (indicated by a

'+' in figures) is placed in high position on the dorsal sheet of the wing membrane. A vein between the two sheets is neutral[6]. The convexity of a vein can vary in its path.

**Wing**: flight body composed of two layers of epidermal cells forming upper and lower layers. It can be thin, membranous or thick, discolored, transparent, or brightly colored, covered with setae or scales. The wing is traversed by an array of ribs and tracheae[6].

### 1.4. Phylogeny of the Ensifera

We follow the molecular phylogeny of Ensifera of Song *et al.*[8]: the Ensifera appear monophyletic and sister group of the Caelifera (grasshoppers and locusts). The Gryllotalpoidea (Gryllotalpidae) are the sister group of the Grylloidea (four families: Mogoplistidae, Trigonidiidae, Phalangopsidae, Gryllidae[9]), forming the clade Gryllidea. The phylogenetic position of the Schizodactyloidea remains unclear, as Gwynne[10] and Jost & Shaw[11] placed them as sister group of the Gryllidea, while Song *et al.*[8] considered them as the most inclusive clade of the Tettigoniidea = [((Hagloidea + Stenopelmatoidea) + Rhaphidophoroidea) + Tettigonioidea].

## S.2. Descriptions of venations of singing Ensifera

### 2.1. Grylloidea Gryllidae (*Oecanthus* sp.) (Fig. 1, suppl. movies 1-2)

Convex veins (A2, A3 and A4) emerge from JB and become distally neutral. AB gives birth to anal vein A1basally convex then distally curved and concave to form the file. AB is situated ventral to JB. All the other veins (Cu, M, R, pectinate concave ScP) emerge from a large basivenal sclerite, which is thus ScB+RB+MB+CuB. Cu is divided into the two branches CuP and CuA. CuP is subdivided into convex CuPb (which extends along A1 and distally ends into a long vein between A1 and CuPa), and into convex CuPa, anterior to CuPb. CuA ends in the basal stem of R+M; M+CuA re-emerges very shortly distally, at the level of the point of

separation of CuPa and CuPb. Note that there is no direct evidence of the presence of the fusion of M with CuA because no independent M is visible at wing base. This fusion can be inferred only after the distal structure of the vein M+CuA identical to the situation in the other singing Ensifera[12]. R is simple in its basal part and joined to ScP to go apart a little basal of the level of the division of CuP. R stays simple and convex over its entire length

Note that, in some other Grylloidea (e.g. the Phalangopsidae *Homoeogryllus orientalis*, the Gryllidae *Xenogryllus eneopteroides* or *Brachytrupes megacephala*) the vein CuPb is vanishing in the area between CuPa and A1, even if it is well visible at its point of emergence from the CuB (data not shown).

**2.2. Gryllotalpoidea** Gryllotalpidae (*Scapteriscus* sp.) (Fig. 1, suppl. movies 3-4)

Two sets of veins emerge from a common basivenal 'AB+JB': the anterior one (anterior anal veins) and the posterior one basally divided into two neutral branches that become distally convex. The most anterior anal vein A1 is the most convex of all these veins. Just distal to 'AB+JB', emerges a relatively concave vein Cu from the sclerite CuB. This stem of Cu divides into a neutral posterior branch CuP and CuA basally fused with the median vein (basally concave then distally convex). CuA re-separates from M a short distance distally as a transverse vein between M and CuPa. CuP is divided into a (basally) neutral CuPb and a more convex CuPa. CuA ends into CuPa. The distal part of CuPb is strongly curved and constitutes the stridulatory file. There is a strong vein that apparently emerges from CuPb as an anterior branch but that Béthoux[12] considered as a possible crossvein. Its exact nature is still undetermined. M+CuA is basally concave, then M is neutral and becomes distally nearly as convex as R. MB, ventral, is independent of the other basivenal sclerites and vein M is fused directly with CuA. The radial stem is joined but not fused to M+CuA and emerges from a

broad basivenal sclerite ScB+RB that also gives birth to ScP. ScP is anteriorly pectinate and strongly convex at its base (contra the situation present in the great majority of the insects).

Between CuP and M+CuA, basal to the fork CuPa – CuPb, a longitudinal structure is observable in the thickness of the wing membrane. It is very thin, looking like a vein but without any relief. This structure does not appear on the tomograms. It is probably a trachea.

**2.3. Prophalangopsidae** Cyphoderrinae (*Cyphoderris monstrosa*) (Fig. 3, suppl. movies 5-6)

Two sets of veins emerge from ‘AB+JB’: the anterior ones (A1 and A2) are convex (anterior anals); the posterior ones are neutral. The most anterior one A1 is also the most convex of these veins. CuB is independent from AB+JB, CuP emerges posteriorly and CuA anteriorly from CuB. CuP is distally divided into two main branches, an anterior concave CuPa and a posterior CuPb that is strongly concave. CuPa is divided into two branches, one longitudinal CuPaα and one transverse CuPaβ. A strongly convex vein “h” is between CuPaβ and distal point of fusion between CuPaα and CuA. The veinlets emerging from CuPb are ending on A1 or on CuPaβ. CuPaα is ending on distal part of CuA. M is visible as a very short vein (see on the cuts in Fig. 3f-h) touching CuA and RB and ending into CuA. M+CuA is strongly diverging from the radial vein and is distally re-separating again into M and CuA. The basivenals of M, R and ScP are fused into a large common basivenal. ScP is pectinate.

The other modern Prophalangopsidae have patterns of venation similar to that of *Cyphoderris*, especially in the CuPb acting as stridulatory file, and the organization of the veins in the area between CuPa and CuPb.

**2.4. Tettigonioidea** Tettigoniidae Phaneropterinae (*Quiva* sp.). (Fig. 3, suppl. movies 7-8)

This taxon has six basivenals AB+JB, CuB, MB, RB, ScB, and CB. Only one vein emerges from each of these basivenals, except for CuB, JB and CB. From AB+JB, emerge

two anal veins that separate from a common stem, one strongly convex A2 that emits numerous veinlets forming a dense and hollow structure ending on posterior wing margin and on the file; the posterior wing margin is basally convex. A1 is convex and distally curved to make the stridulatory file. The file is strongly inflated and bears the series of small teeth ventrally. A vein looking like an anterior branch of A1 that separates from the base of the stridulatory file and forks distally. Béthoux[12] considered it as a crossvein named column. CuA and CuP emerge from CuB. CuPb is absent. CuP(a) is a weak and simple vein that ends in distal part of CuA, maybe to give birth to the branches CuPaα and CuPaβ, but these last veins are not clear in *Quiva* sp. From MB, M is directly emerging very basally to end into ScB+RB, and more distally goes into CuB. M is visible for a very short length between RB and CuB (see on the cuts in Fig. 3n). M+CuA is straight, making a relatively open angle with the radial vein. M and CuA are re-separating distally. The radial vein is straight, basally simple. ScP is basally simple and ScB fused to RB. CB is joined and fused very basally to RB+ScB. From CB emerges two costal veins.

**2.5. Mesoedischiidae** Gorochov, 1987 (Fig. 5a)

The Triassic male *Mesoedischia obliqua* Gorochov, 1987 has a concave stridulatory file (see Fig. 5a), emerging from a convex vein that also emits a convex anterior branch at the base of the file. If it was the vein CuPb, it should have been concave and not convex. Thus we propose to interpret it as the vein A1 that has a concave broad posterior branch that bears the file and a convex anterior branch. The concavity of the vein interpreted as the stridulatory file is coherent with this function as the file has to be rubbed by the other tegmen. The branches of CuP are also relatively concave. As *Mesoedischia* shares a complex apomorphic character with the Tettigoniidae, it probably belongs to the stem group of the Tettigonioidea. *Mesoedischia* differs from the modern Tettigoniidae in the presence of a well-developed CuP

with branches CuPa and CuPb (plesiomorphic state of character). CuPb is concave, CuPa is basally fused with M+CuA and re-emits its branches distally.

**2.6. Liassic Grylloidea** (Fig. 5b, Supplementary Fig. 3)

One of us (MH) discovered a cricket tegmen (specimen MNHN.F.A57514) in the Lower Toarcian of Luxembourg (Bascharage), in a limestone lens. The veins are pyritized and some traces of color are possibly preserved. We observed the venation under normal binocular microscope with alcohol and under an environmental scanning electron microscope (ESEM), using the Back Scattered Electron (BSE) mode to detect the chemical nature of the specimen. The presence of iron oxides was verified under the mode Energy Dispersive X-ray Spectroscopy (EDS). Despite the absence of the extreme base of the fossil tegmen, we observed a very short and weak longitudinal vein between the file and the cubitus posterior (Figs. S10C, S11, S12, S13). This vein is exactly in the position of the CuPb of the Grylloidea that vanishes in the area between CuPa and A1. The presence of this vein implies that the file is on vein A1. The small denticles of the file are clearly visible. Thus, this tegmen corresponds to that of a Grylloidea. Nevertheless, it has a short vein h and a CuPaβ that separates from CuPaα very close to the point of fusion with CuA and base of vein h, which somewhat reminds what exists in the Prophalangopsidae. This tegmen shows a large lanceolate cell between R and M, characteristic of the Protogryllidae Zeuner, 1937, a Mesozoic family with oldest representatives dating in the Lower Jurassic. So far as we know, no clear synapomorphy between the protogryllids and the modern Grylloidea *sensu stricto* was proposed in previous publications. The present study compensates for this lack and brings an original proof for the presence of Grylloidea in the Lower Jurassic.

**2.7. *Liassophyllum caii*** (Fig. 2)

Gu *et al.*[13] described *Liassophyllum caii* from the Middle Jurassic of China, on the basis of a series of specimens. These authors considered it belongs to the Haglidae Cyrtophyllitinae. The holotype is a well-preserved tegmen. New photographs, kindly provided by our colleague Jun Jie Gu, clearly show that between CuPa and the file, there is a short vein that has the same base as CuPa, runs towards the file, touches it and vanishes distally in the area between CuPa and the file. This vein typically corresponds to the weak and short CuPb as observed in modern true crickets. Thus, the file is on the vein A1. The situation is quite similar to that of the Grylloidea and the Liassic fossil described above. This tegmen is of great interest because the structure of the distal part of the stridulatory structure is of prophalangopsid type, unlike in the Liassic fossil described above and in the modern Grylloidea. It confirms that some taxa of the fossil record present character states homologous to that of the prophalangopsid-type and other homologous to the grylloid-type of stridulatory apparatus.

**References**


1. Boistel, R. *et al.* Shake rattle and roll: the bony labyrinth and aerial descent in squamates. *Integrative Comparative Biology* **56**, 957–968 (2011).
2. Lauridsen, H. *et al.* Inside out: modern imaging techniques to reveal animal anatomy. *PLoS ONE* **6**, e17879. (2011).
3. Zanette, I. *et al.* Looking inside marine organisms with magnetic resonance and X-ray imaging. In Imaging marine life (ed. EG Reynaud), pp. 122–184. Weinheim, Germany: Wiley-VCH Verlag GmbH & Co. KgaA. (2013).
4. Kukalová-Peck, J. Origin and evolution of insect wings and their relation to metamorphosis, as documented by the fossil record. *Journal of Morphology* **15**, 53–126 (1978).

5. Kukalová-Peck, J. Origin of the insect and wing articulation from the arthropodan leg. *Canadian Journal of Zoology* **61**, 1618–1669 (1983).
6. Séguy, E. Introduction à l'étude morphologique de l'aile des insectes. *Mémoires du Muséum National d'Histoire Naturelle*, Série A, *Zoologie* **21**, 1–248 (1959).
7. Desutter-Grandcolas, L. Functional forewing morphology and stridulation in crickets (Orthoptera: Grylloidea). *Journal of Zoology* **236**, 243–252 (1995).
8. Song, H. J. *et al.* 300 million years of diversification: elucidating the patterns of orthopteran evolution based on comprehensive taxon and gene sampling. *Cladistics* **31**, 621–651 (2015).
9. Chintauan-Marquier, I. C. *et al.* Laying the foundations of evolutionary and systematic studies in crickets (Insecta, Orthoptera): a multilocus phylogenetic analysis. *Cladistics* **32**, 54–81 (2016).
10. Gwynne, D. T. Phylogeny of the Ensifera (Orthoptera): a hypothesis supporting multiple origins of acoustical signaling, complex spermatophores and maternal care in crickets, katydis, and weta. *Journal of Orthoptera Research* **4**, 203–218 (1995).
11. Jost, M. C. & Shaw, K. L. Phylogeny of Ensifera (Hexapoda: Orthoptera) using three ribosomal loci, with implications for the evolution of acoustic communication. *Molecular Phylogenetics and Evolution* **38**, 510–530 (2006).
12. Béthoux, O. Grylloptera - a unique origin of the stridulatory file in katydids, crickets, and their kin (Archaeorthoptera). *Arthropod Systematics & Phylogeny* **70**, 43–68 (2012).
13. Gu, J.-J., Zhao, Y.-Y. & Ren, D. New fossil Prophalangopsidae (Orthoptera, Hagloidea) from the Middle Jurassic of Inner Mongolia, China. *Zootaxa* **2004**, 16–24. (2009).

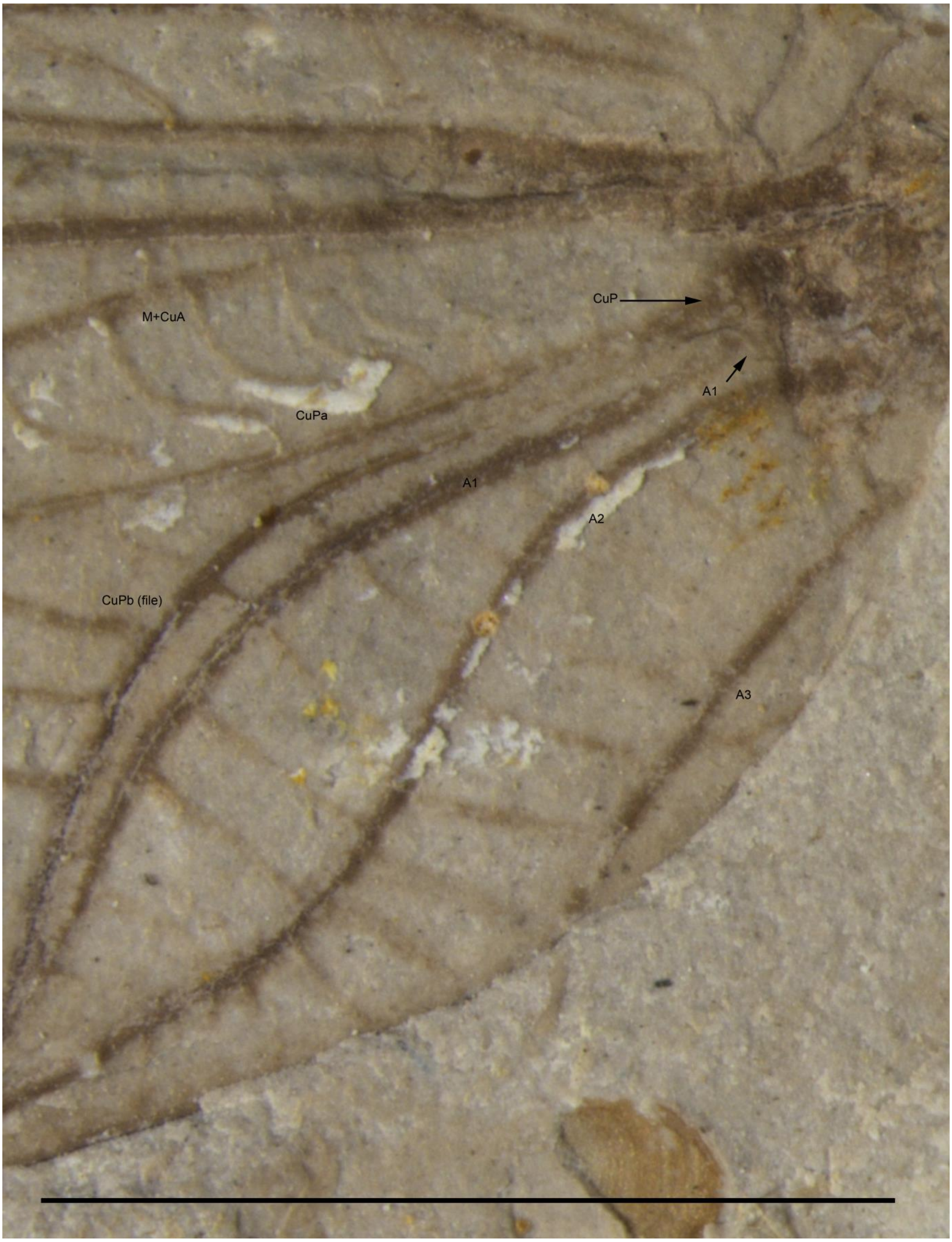


**Supplementary Figure 1 | *Sigmaboilus peregrinus* Gu *et al.*, 2009 (paratype CNU-ORT-NN2008041P)**. Wing base. (scale bar represents 5 mm) (copyright Dong Ren).

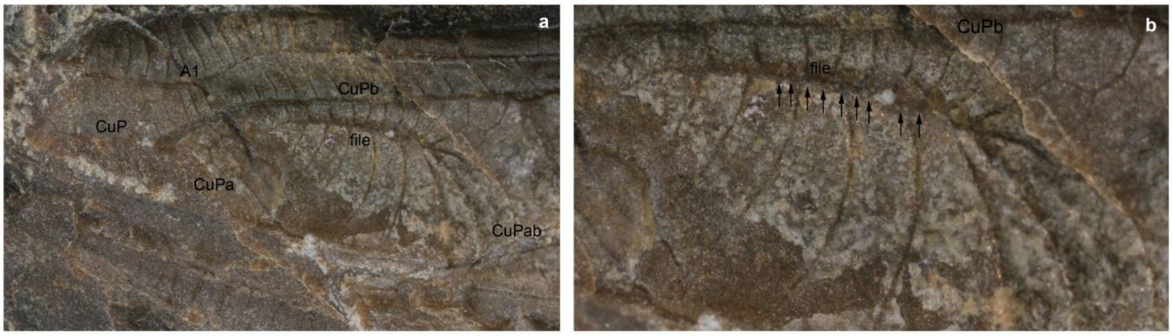


**Supplementary Figure 2 | a-b. Caloneurodea Permostridulidae (*Permostridulus brongniarti*, holotype Ld LAP 499ab, imprint).** (**a**). Wing base. (**b**). Detail of the file (arrows indicate the teeth that are visible as small conical holes) (copyrights R. Garrouste).

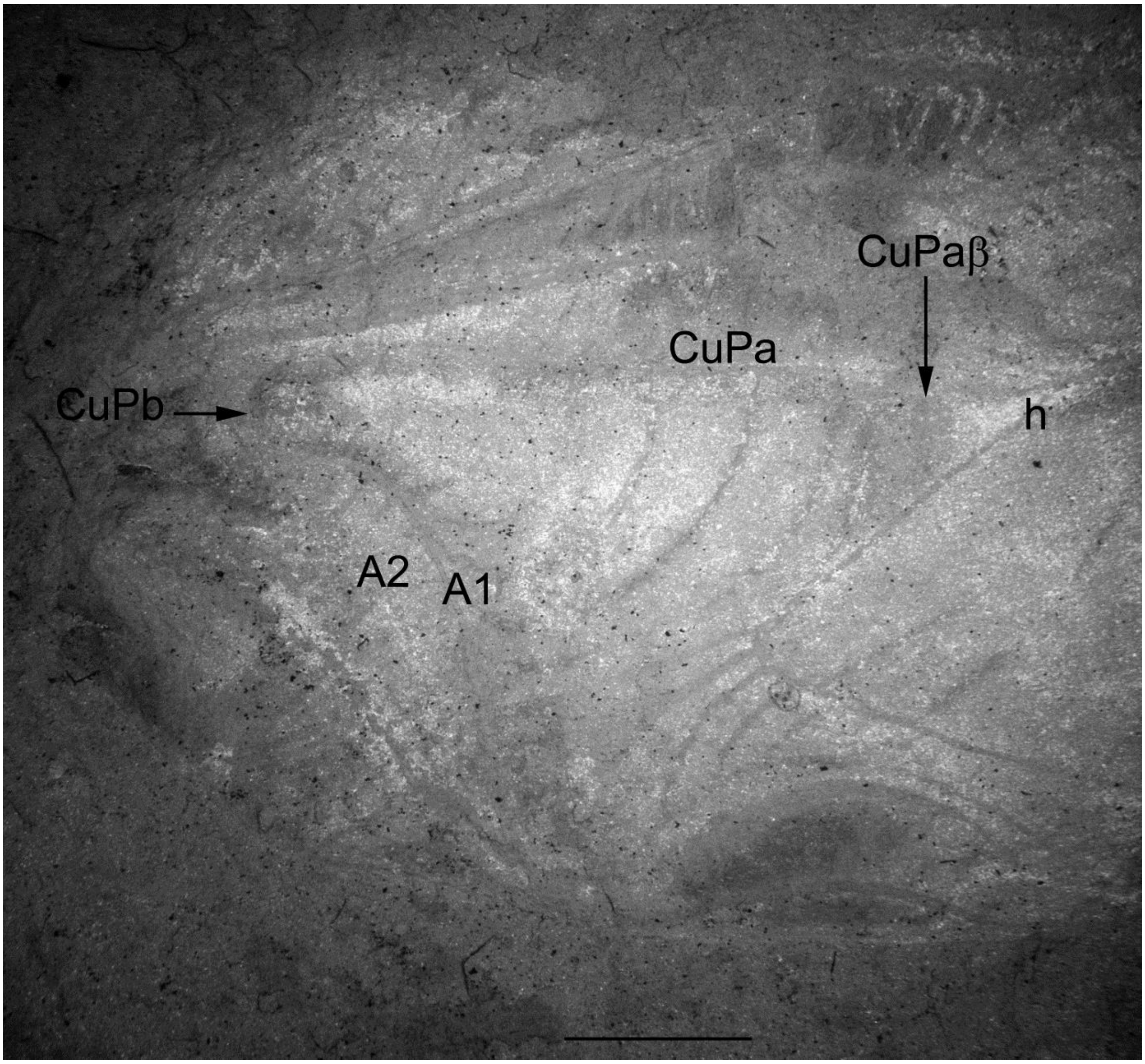


**Supplementary Figure 3 | Grylloidea indet. (Specimen MNHN.F.A57514).** Torcian, Bascharage, Grand-Duché du Luxembourg. (scale bar represents 2 mm) (copyright R. Garrouste).

**Supplementary movie 1 | *Oecanthus* sp.** Movie showing the course of the veins, without colors for interpretation.

**Supplementary movie 2 | *Oecanthus* sp.** Movie showing the course of the veins, with colors for interpretation.

**Supplementary movie 3 | *Scapteriscus* sp.** Movie showing the course of the veins, without colors for interpretation.

**Supplementary movie 4 | *Scapteriscus* sp.** Movie showing the course of the veins, without colors for interpretation.

**Supplementary movie 5 | *Cyphoderris monstrosa*.** Movie showing the course of the veins, without colors for interpretation.

**Supplementary movie 6 | *Cyphoderris monstrosa*.** Movie showing the course of the veins, without colors for interpretation.

**Supplementary movie 7 | *Quiva* sp.** Movie showing the course of the veins, without colors for interpretation.

**Supplementary movie 8 | *Quiva* sp.** Movie showing the course of the veins, without colors for interpretation.
